# Personalized Gamification for Learning: A Reactive Chatbot Architecture Proposal

**DOI:** 10.3390/s23010545

**Published:** 2023-01-03

**Authors:** Carina S. González-González, Vanesa Muñoz-Cruz, Pedro Antonio Toledo-Delgado, Eduardo Nacimiento-García

**Affiliations:** Departamento de Ingeniería Informática y de Sistemas, Universidad de La Laguna, 38200 San Cristóbal de La Laguna, Spain

**Keywords:** open learner modeling, gamification, chatbots, personalization, game learning analytics, user modeling

## Abstract

A key factor for successfully implementing gamified learning platforms is making students interact with the system from multiple digital platforms. Learning platforms that try to accomplish all their objectives by concentrating all the interactions from users with them are less effective than initially believed. Conversational bots are ideal solutions for cross-platform user interaction. In this paper, an open student–player model is presented. The model includes the use of machine learning techniques for online adaptation. Then, an architecture for the solution is described, including the open model. Finally, the chatbot design is addressed. The chatbot architecture ensures that its reactive nature fits into our defined architecture. The approach’s implementation and validation aim to create a tool to encourage kids to practice multiplication tables playfully.

## 1. Introduction

Learning platforms are widely extended in educational institutions. Despite being very useful for organizing content, providing links to external resources, evaluation management, facilitating communication among students, etc., they also have essential features to achieve better learning outcomes. One of these features is improving learned motivation and engagement. In this sense, introducing gamification into the learning platforms might be a crucial factor. The gameplay elements might give the students a better understanding of progress, accomplishment, and community among them. Nevertheless, it is well known that not all students are alike, and the approach’s effectiveness might be compromised if a one-fits-all strategy is fixed for the gamification implementation.

Personalized gamification enables a new ecosystem, providing an intimate experience to users to increase the application’s effectiveness when motivating certain behaviors. The trigger comes from the new possibilities of technology toward an interconnected society and the evolving nature of work in the information age [1].

Some authors [1] have analyzed gamified system user models. In addition, different types of players, ways of having fun, and motivations for playing have been identified [2]. The authors have simplified user modeling, the key to the customization of a system, by proposing a taxonomy of players. In this way, it is possible to offer user models that are adaptable and customizable to the types of players. Some of the newest frameworks [3] consider applying the Gamification Hexad User Type model [4], where six user types are described: socializers, free spirits, achievers, philanthropists, players, and disruptors. Thus, in gamified learning systems, the student model must represent how people play: its player types.

Moreover, conversational agents, or chatbots, provide a natural language interface to users. They are familiar with intelligent educational systems [5]. Nowadays, chatbots are a promising intelligent tool to assist us in many different ways and can be used for personalized gamified learning systems.

In the past, pedagogical agents or avatars have been used for tutoring the individual learner and helping with learning tasks. To improve the effectiveness of the gamification of the learning tasks, this paper proposes using chatbots to personalize feedback for the contextual gamified learning experience.

Through presenting information in different ways and providing opportunities for discussion, the learner experience can be enhanced with a conversational system [6]. In this sense, a chatbot can be integrated into an open system where the dialog system is a module within the learner model.

This paper describes a proposal to represent an open student–player model and different techniques to provide personalized feedback through several platforms using chatbots.

The main contributions of this paper can be summarized as follows: The paper proposes a reactive chatbot architecture, which allows personalized gamification for learning and a prototype validation of the Proposal. The Proposal includes, firstly, a joint open player–student model of the users. Secondly, the general architecture of the system is addressed. Thirdly, a Chatbot Reactive Architecture is described. Finally, it is shown the integration into a Gamified Learning System.

This paper is structured as follows: Section 2 describes state of the art concerning related fields, followed by Section 3, in which related work is analyzed. Our Proposal is detailed in Section 4, and the validation of the system is included in Section 5. Finally, Section 6 presents the conclusions and further work. 

## 2. State of Art

In this section, we will present the state-of-the-art fundamental topics involved in this work. First, we offer the basic concepts related to user-adaptive systems. Then, we give the fundamentals of gamification and game-based learning. Then, game user models are presented with information on how to measure them through analytics. Finally, chatbots are offered as a solution for natural interaction and conversation with users.

### 2.1. User-Adaptive Systems

User-adaptive systems can adjust their interfaces and contents to features gathered in user models to enable a system to behave personalized for different users [7]. The user model is a student or learner model in the educational system’s domain. Having a better understanding of the user, adaptive designs can provide better content, customization, and adaptation of the interface.

To acquire information about users, several methods can be used. It can be achieved directly through an initial questionnaire, user interaction in the form of specifically designed quizzes or games, etc., or indirectly with assumptions about users based on their interaction behavior [8]. Methods can include rules to predict features or assign different users to predefined groups with known features—for example, the methods of probabilistic reasoning taking evidence from various sources. The purpose of plan recognition methods is to link individual user actions to goals. Machine learning methods aim to detect patterns in user actions and use those patterns to predict future actions [9,10]. Grouping methods allow the generation of user stereotypes by grouping users with similar behaviors or characteristics. The user’s data, which are interesting for personalization purposes, can include data about the user (knowledge, preferences, skills, etc.); usage data, such as selections in applications or webpages, behavior, purchases, user ratings, etc.; and environmental data, such as software and hardware environments, current location, etc.

Usually, personalization occurs separately within each system. However, there are disadvantages to using isolated personalization approaches, such as the time invested by a user for the personalization of a system is not transferable to others, or users need more control over the information, etc.

### 2.2. Gamification and Game-Based Learning

Gamification uses game dynamics, mechanics, and other game elements in nongame systems to motivate the students. For example, challenges, badges, levels, rewards, customization, etc. However, we must take into account Gartner’s warnings. Many solutions based on gamification fail because they were created without a formal design but by adding elements without connection or coherence instead [11,12].

There are different game mechanics, such as those that focus on human behavior, those that are related to feedback, and those used to structure achievements and skills and show their progress in the game. Likewise, other mechanics can be applied to gamification in education, such as time constraints, content exploration, unlocking, and collaborative and competitive challenges, in addition to other game elements that allow for achieving goals and rewards [13,14].

However, more than this may be needed for students to learn. Game-based learning occurs when the game can result in training new skills when learning comes from the game [15,16]. Students can explore games in a learning context designed by teachers, so teachers and students can collaborate on the experience of playing a game. In game-based learning, the theoretical contents are presented utilizing a video game and can be combined with simulation to create a serious game [17,18,19]. Using simulation makes it possible to create real situations so that users can practice their skills.

Some characteristics that define game-based learning are that learning must be carried out using engaging scenarios, creating a positive experience and that there must be evolution (for example, through overcoming obstacles or levels) [20]. Establishing a model that can be used in designing and analyzing educational games is necessary. The primary purpose of the model is to connect gameplay with learning. According to Yen-Ru Shi and Ju-Ling Shih [21], the model should include the game goals, teaching objectives, and the experience that will be provided to the players.

The game should increase the level as the player progresses, providing challenges to motivate while teaching. Consequently, the game has to adapt to the player’s characteristics since not all players are equal, nor do their skills evolve simultaneously. Still, this adaptation must always be transparent to the player, and it should keep the conceptual model and the user interface and avoid any usability problems.

Some advantages of gamification and game-based learning are:

- Using games can improve the motivation and engagement of learners. Games include rules, goals, objectives, etc., that offer interactive experiences that motivate and allow students to experiment, improve decision-making, and plan their actions.
- Learners can obtain immediate feedback about whether they made a good decision while playing. Additionally, they can experience the long-term effects of their decision-making. Furthermore, learners can experiment in a safe environment.
- Gamification and game-based learning environments can obtain higher retention rates than other learning methods. This happens because learners have to remember the rules, use strategic behavior, think quickly and creatively, plan future moves, etc.
- Gamification and game-based learning also allow to development of physical skills, cooperation, teamwork, spatial skills, etc.

While game-based learning is a type of gameplay with defined learning outcomes, gamification consists of just adding games to learning objectives. It utilizes rewards and motivation to attract and engage individual participants [22].

### 2.3. Learner/Player Models

The student model includes not only all the static attributes and information about the student, such as preferences, learning style, age, previous knowledge, etc., that represent the student profile, but also dynamic attributes, such as user records, history, etc., obtained through the student interaction with the system. Additionally, a formal definition provided by Machado et al. [23] says that player modeling is “an abstract description of the current state of a player at the moment”. The objective is to improve aspects such as adaptability, playability, challenges, etc., by the use of the model of characteristics and behaviors of players during the game. Montserrat, Lavoue, and George analyze the design of user models and personalized gamification in gamified systems. The problem is how to orient and guide learning since each player can have a different approach and attitude. In several educational studies, it is possible to find opinions about types and learning styles and how it influences learning outcomes [24,25,26].

Bartle proposed in 2006 a taxonomy of player types which consists of four characters: Achievers, Explorers, Socializers, and Killers. Heeter and Winn [27] proposed another taxonomy of player types for learning games, including gender differences, classifying players as Competitive, Engaged, Careless, and Lost. Kim [28] created a taxonomy of player types focused on casual games based on Bartle’s model. Likewise, other authors and [3,29] have evaluated player typologies based on demographic factors. Another model is the Hexad User Types [30], which defines gamers: Socializers, Free Spirits, Achievers, Philanthropists, Gamers, and Disruptors.

Generally, the player’s profile can be analyzed with explicit (questionnaires or tests) or implicit methods (including in the game).

Finally, an open learning model allows showing the student’s learning status, knowledge, errors, and difficulties, among other factors that affect their learning [31,32]. Students can see their learning status, and this advantage provides opportunities for feedback on their knowledge and stimulates metacognition [33].

### 2.4. Game-Learning Analytics

Since video games have become an apparent reference in the entertainment industry, there have been initiatives to combine their power to improve education. However, many questions arise about the matching of both disciplines. On the one hand, education is quite challenging, and a deep understanding of how students learn is crucial to lead its development. Learning analytics has become popular as an attempt to use data-driven support in education. Rich data representation and visualization, information retrieval, recommendation systems, data mining, and machine learning are just some of the fields involved in learning analytics activities. On the other hand, game analytics, which includes research studies about games, players, motivations, roles, models, etc., has been developed very recently.

Both challenging games and learning gamification are clearly and naturally strengthened when similar analytics from both disciplines are introduced. The combination of these fields has led to what has been called Game Learning Analytics [34]. From a research perspective, the main goals of this discipline are to increase the understanding of how students interact with the games, provide tools to understand the educational impact of fun on the students, and even reduce the development costs of digital games.

Furthermore, from a purely educational perspective, many other goals can be established: prediction of student performance, game experience personalization, reducing student dropouts, etc. [35].

The main components of G.L.A. can be categorized into instrumentation, which recovers information on the game side about the user interaction; collection and storage on a server; real-time analytics, for immediate feedback and maximum effectiveness; batch analytics for long-term decision; dashboards and Key Performance Indicators (KPI), for educators to follow their student’s outcomes; and stakeholder decision making.

Systematization methodologies for G.L.A. have been recently proposed to reduce the costs of serious game development and study [36]. This includes using standard tracking models to exchange information between the game and the analytic platform and using standardized analysis and visualization assets.

### 2.5. Chatbots

Chatbots are currently experiencing an explosion in their integration into real applications. Conversational interaction is one of the most natural ways humans have developed to transmit information. The development of artificial intelligence, speech recognition, and natural language processing has passed the acceptable usability threshold, and therefore chatbots can now be found in all kinds of commercial applications [37]. Firstly, video games have traditionally integrated chats as a form of communication among human and non-human players. Secondly, educational frameworks require continuous feedback to the students to guide them through the learning process, not only to transmit new information, answer emerging questions and make an evaluation, but also to motivate and enhance students along the process.

## 3. Related Works

In this section, related works to gamification and chatbots will be presented, Chatbots are applied to education, and their architecture will be described.

### 3.1. Elements for a Personalized and Contextual Experience

Most gamified systems use PBL (points, badges, and leaderboards) to motivate or try to change user behavior [38]. Although gamification works in the short term, the same does not happen in the long term due to the difficulty of keeping the user motivated and engaged over time. One way to solve this problem is the personalization of gamification. By understanding users’ personalities, emotions, behaviors, and actions, a better experience can be provided to the end user. Therefore, in a gamified experience, communication with the user must be adequately related to the characteristics of the user, context, and events.

On the other hand, social gamification can also be carried out, including communities, social networks, and using big data. The new generation of gamification systems includes the ability to personalize the user experience and its context. Taking into account the characteristics of the new generation of gamification systems, in this article, we will see some aspects that must be considered for its design for educational purposes.

Different elements can be identified in a personalized gamification system for learning [38,39]: types of player–learners, static and dynamic user attributes, transactions, activity tracking, ratings, observable user behavior, and behavioral determinants.

Additionally, the main objectives of an adaptive game-based learning system are to improve interactive communication and motivate the player. Adaptation must be oriented on player characteristics. The transformation must be oriented to the features of the player, so it must follow a scheme to adjust the game as necessary: with constant interaction, storing the information of the game and the player in a database, adjusting the parameters, levels, events, etc., of the game, evaluating the progress of the player, and adapting accordingly. The advantage is that they are built to adapt to new situations, and if a player changes the game’s style, the system adapts consistently [40,41].

Machine learning techniques can be useful for personalization and adaptation. Some authors have reviewed intelligent adaptation methods used in serious games for education, finding that the most used were rule-based, Bayesian, fuzzy, and reinforcement learning [42]. Another related technique to personalization in serious games was incorporating dynamics of learner behaviors as learning attributes in a Petri net model for knowledge reasoning and learning [43]. Tang et al. [44] describe an intelligent component based on the k-nearest-neighbor (kNN) classification method to provide personalized feedback and guide the student through the learning process.

### 3.2. Chatbots Architectures

There are two main types of chatbots for business and entertainment. The goal of an entertainment chatbot is to have a conversation with the user. The chatbot’s effectiveness is generally determined by how much time the user talks with the system (daily, average duration in a period, etc.). Alternatively, business chatbots are usually designed for specific goals. They can be classified into support chatbots, assistant chatbots, and skills chatbots.

The most common models or chatbots architectures [45] are the following:

- Heuristic models use rules, classifiers, and other AI techniques. They are used mainly in entertainment chatbots to select answers for the user among the multiple options available.
- Generative models which train the chatbot with deep learning techniques. They are complicated to develop since they need many examples for learning, and even then, they can generate generic or incoherent answers.
- Selective models with machine learning and classification algorithms. As in the previous case, they need many examples to find patterns in datasets.
- Retrieval-based models use previous messages and conversation context to choose the response from a message list. They are more predictable and easier to develop.
- Architecture with response selection, in which the chatbot can display the same message in different ways, adapting responses to the user using previous chats and metrics (usually in business chatbots, such as purchases and sales, customer satisfaction, etc.).

### 3.3. Chatbots in Education

The literature on chatbots in education and games for learning is limited. There are few studies on gamification and conversational AI in education [46]. For example, Lin et al. [36] related a platform for collecting, evaluating, and annotating human–chatbot interactions. Ahmed Fadhil and Adolfo Villafiorita [47] have developed an educational chatbot to advise and teach about healthy lifestyles using a game called ‘CiboPoliBot’ Some applications such as Rosetta Stone and Duolingo [48] use chatbots to teach foreign languages. For example, Duolingo is a bot and the creation of a Pittsburgh startup, launched in 2011, which has 150 million users. It includes gamification with rewards and goals for users. Other examples are Jill Watson, a teacher’s assistant at the Georgia Institute of Technology, whose true identity is an I.B.M. AI system, and the Chatbot Campus Genie, which will be able to answer questions for students about life on campus at Deakin University (same as in the previous case, the intelligence comes from I.B.M.’s supercomputer system). In 2017, the first Moodle chatbot was shown in MoodleMoot Australia. Its objective was to help the user by offering search services. In the future, it is expected that it incorporates more context factors, evolves towards “Natural Language Understanding (N.L.U.)”, and identifies emotions and interacts with the user consequently [49].

## 4. Problem Definition: A Reactive Chatbot Architecture

The main problem of designing a reactive chatbot architecture with gamification is the mutual fulfillment of many requisites of different natures. Some of them are already present in existing solutions. Nevertheless, the difficulty of building an architecture that might support them successfully simultaneously is still a challenge.

Those requirements can be grouped into three main categories:

Adaptability: The system behavior should not be fixed independently of the context, user nature, educational objectives, motivational status, etc.

A1. Reactive: In the short term, natural interactions that humans are used to are highly reactive to the context. The system architecture should be able to adapt its response promptly and with precision to changes in user inputs.

A2. Customizable Goals: With a broader time horizon with respect to the previous requirement and from the perspective of the educator’s objectives for the learner or user, it is important that the system allows the customization to meet the specific needs and goals. 

A3. Experience Personalization: From the learner’s perspective, the architecture should also allow for personalizing the learning experience. For this goal, the system might need to use data related to past performance, interests, and goals.

A4. Autonomous adaptive learning: Finally, from the system perspective and also in a broader time horizon with respect to the Reactive requirement, the architecture should allow the system to adapt dynamically to the learner’s abilities. In order to achieve this, it should be able to provide customized content and feedback based on its progress and performance.

Motivation: Improving user motivation has been mentioned as one of the key objectives and requirements for the architecture.

M1. Gamification elements: The architecture should incorporate a gamification engine with support for gamification elements. Since users might have different goals and interests, these elements should be adaptable.

M2. Social features: Social interactions are a key part of student’s motivation. The architecture should allow the incorporation of social features. Among these features is the ability to communicate, cooperate, or compete with other students or groups. These features are essential to developing a sense of community and support among learners.

Universal: In order to attend to the differences among users and situations, the system can adapt and react to a great diversity of factors. The natural consequence of that is that such a system design should incorporate features for its universal use.

U1. Scalability: In terms of the number of users, the system should be able to deal with a growing number of simultaneous users and interactions with the system.

U2. Integration: The system should be designed to be easily integrated with systems and platforms, such as LMS or CMS.

U3. Data privacy and security: The users’ personal and profound profiles will be systematically stored in the system. The risk of unauthorized access to the information should be specifically incorporated into the design, as well as the assurance that the system is compliant with relevant regulations and laws.

## 5. Proposal of the System Model

Our Proposal for a modular and intelligent chatbot will be presented in this section. First, we will describe the open model student and the player model due to the design and plan to apply the chatbot in an educational context with a gamification approach. Then, we describe our architecture for a chatbot, with several components for intelligence, interoperability, and easily maintain due to its modularity.

### 5.1. Open Student-Player Model Design

One of our Proposal’s challenges is presenting a joint model for the user. In this case, the user would integrate components from two roles simultaneously, a student and a game player. Both parts of the model have a static model of the user or profile and a dynamic model that should be continuously updated.

An open model’s primary goal is to keep the user knowledge state updated. This state may be used for different purposes. Systems behavior and personalization would depend on this state, being this, therefore, the most direct application. Additionally, there are other desirable features for the use of the model, such as user awareness [50]. There is a bibliography regarding the advantages of including understanding the systems to obtain benefits at the level of usability. For example, Collazos et al. [51] propose a taxonomy and define steps and stages to guide the design and implementation of awareness in collaborative systems. In addition to various mechanisms, different collaboration methods make it necessary to identify and characterize them correctly. Understanding issues such as misconceptions, skills, limitations, preferable activities, etc., has boosted performance since the user can focus on strengthening weak skills and exploiting comparative advantages when needed to overcome difficulties.

The challenge, therefore, is not to build the model itself. There have already been defined, for example, categorization of types of learners, categories for players, and the main features to describe them, as has been mentioned before. Adding both features is the most straightforward approach to building a model in our context. Nevertheless, this could lead to severe problems in terms of practical implementation. It is well known that modeling is the art of designing incorrect simplifications of a complex reality, but simplicity leads to making the correct decisions. Each additional feature incorporated into our user model increases its complexity and harms its usability.

From an information perspective, as well as in the machine learning domain, this problem is known as the curse of dimensionality. Firstly, for each extra dimension, the number of possible instances and types of students, in this case, increases exponentially. To update the model, the information needed grows exponentially as well. This is a substantial drawback since the information that can be gathered from users is numerous but finite. Therefore, limited information limits the accuracy of the induced model. Secondly, many activities are available for the system to match an exponentially large number of users.

Consequently, having such a complex user model is not justified at first. Finally, user awareness may be compromised with complexity. Simple models might be more inaccurate than complex ones, but they can draw a clearer picture for human comprehension. As mentioned above, the real world is the best model, but still, we need models and simplification to boost performance in decision-making.

As a consequence of the stated problem, an automatic model generation based on machine learning techniques is proposed. In machine learning, Principal Component Analysis is a well-known technique to reduce the dimensionality of a problem. The main idea behind this is to perform a statistical analysis of data points from a large dimension distribution, measuring which dimensions can better explain the data variation. Projecting the original data on these dimensions would lead to significant data compression, ensuring minimizing the information loss along the process. Information gain or entropy are some of the basic statistical measures used for this purpose.

Our user model, therefore, is dynamically generated as users interact with the system. Such an approach is a clear advantage for a method used across multiple platforms. Incorporating additional features to the model or drifting the model design to be more accurate in a new context is now an inherent feature. Nevertheless, there is a clear disadvantage since the model dimensions themself could be modified. This means that no static set of rules could be applied to exploit the user model to choose the proper actions of the system.

To solve this issue, some methods from the machine learning field might be applied. Two approaches are proposed. Firstly, using techniques of reasoning under uncertainty solves the problem. The entire model, before the dimensionality reduction, is considered. The variables discarded from the model for the decrease in dimensionality are considered latent variables, and the ones still in the model are observables. Consequently, the simplified model is still usable for the system action selection.

A second and completely different approach is to assume a Reinforcement Learning environment for the model exploitation. Since not all simplified models may be foreseen beforehand, they are considered unknown. The system, therefore, should improve itself with user interaction, using an exploration–exploitation tradeoff strategy. The idea is to dynamically explore different actions on different students’ profiles and gather feedback about the results. Good results would encourage the selected association, and on the contrary, bad results would discourage it. All this should be undertaken while minimizing the users’ experience until the system is fully trained. This is precisely what the proposed Reinforcement Learning techniques accomplish in our scenario, exploiting the results of previous experience when the uncertainty about the model behavior disappears but exploring other possibilities while the uncertainty is still high.

As a result, the proposed model is highly flexible and adaptive but still a simple model which can dynamically be updated, not only with new categories for the existing dimensions but also with new ones. That, therefore, is a crucial advantage for systems designed to operate in multiple digital spaces.

Although motivation and gamification of the learning process are critical, it must be taken into account that designing highly interactive scenarios could have some negative effects. Technology addiction, either by the Internet, mobile phone, social media, etc., has emerged and is increasing due to the use of technology daily. There are recent studies have been carried out to show that this addiction can distract from other activities, affect socialization, sleep, etc. [52,53,54]. For example, Yang [55] studied the harmful effects of online gaming and how it affects real-life interpersonal interactions.

On the other hand, other chatbots, such as OpenAI GPT3 chat, are being used for multiple purposes, one of them being therapeutic [56], without being validated for this purpose, which could be a risk for people with mental health problems. However, in educational subjects or business environments, for example, they can support multiple tasks for learning or solving doubts. In the case that we propose, we do not consider the effects to be similar to an addiction to games; since as they are learning activities, the motivation of the students is necessary, but there should not be a negative influence from the social point of view, etc. [57].

### 5.2. Architecture Proposal

Considering the elements mentioned previously, we can define the main components of the personalization process in gamified learning systems.

(a) Open Player–Student Model: An open student model permits the students to observe and reflect on their progress and consists of a set of features visual and interactive that show data of progress, knowledge, or other statistics of the activity of the student. [58,59]. The learner model is defined through static attributes like learning style, preferences, preferences, etc., (profile) and dynamic, such as the state of knowledge on the topics (history, user records). It comprises the student profile (persistent information such as cognitive age or disability) and student records (data collected through interaction with the system). The student model is responsible for generating the student’s knowledge state. The student’s knowledge status is related to topics (concepts, facts, procedures, rules, skills, etc.), misconceptions (well-understood errors, error library, incorrect knowledge with errors, as well as missing knowledge), skills learning (learning style, preferences, habits, type of thinking (inductive or deductive), and degree of concentration), affective skills (commitment, challenge, boredom, and seriousness), student experience (user history, student attitude, and experience), and stereotypes (general knowledge of the student and initial student model). To this student model, we can add the characteristics of the player model, creating a player–student model. This model will allow us to identify the player’s preferences and determine the user’s commitment to the proposed activity, and we can use the Bartle or Myers–Briggs player types.
(b) Game learning analytics and personalization modules: Learning analytics is used to analyze the user’s interaction with educational purposes, for example, how the learners use the domain knowledge in an intelligent tutoring system and evaluate student progress. Combined with game analytics to collect and analyze game user interactions, game learning analytics allow for improving the use of educational games. Furthermore, using many platforms with user data for analysis through synchronous and asynchronous modes allow prediction, personalization, adaptation, etc. This user data can predict students requiring attention and supporting interventions, motivation and retention, tracking, and feedback.
(c) Contextual information module: The contextual information module gathers information regarding the user’s location (school, home, or outside), the activity they are performing (free time, school time, etc.), the type of device they are using (laptop, smartphone, tablet, or desktop computer), and other contextual information related to the activity (virtual activity, in the classroom, collaborative action, etc.). The user interface’s gamification functions must be adjusted to various contexts by the gamification engine.
(d) Gaming tracing: This module deals with activity monitoring, observable user behavior, and the factors influencing a user’s behavior. The user data gathered during system engagement are examined to design a unique gamification system. As a result, interface functionality is adjusted to account for user behaviors.
(e) The module’s gamification engine combines game mechanics with game dynamics to drive engagement, increasing the experience. Thus, game dynamics guides how players interact during the run-time. So, game dynamics are the procedures and rules of a game related to the level of data representation and game algorithms.

### 5.3. Integration of a Chatbot Reactive Architecture in Gamified Learning Systems

As has been described before, the integration of chatbots in gamified systems seems both natural from the user perspective and challenging from the research and development point of view.

A chatbot in a gamified learning system must be able to integrate different roles. It is supposed to lead the user learning process in the most classical and academic approach helping with tasks and activities. At the same time, it should motivate and enhance the student in the process.

The chatbot should be able to use different sources of information. Academic activities are just one of them. The game interaction is also a rich data source, as gaming analytics has proven in recent years. Furthermore, mobiles, wearables, and personal assistants also provide continuous activity information from the students that might be interestingly integrated.

The interaction interface is a challenge in itself. The reactive architecture is designed to answer the user reacting to his events, using contextualized information, but also allows it to initiate dialogs when needed proactively.

Based on the previously described general architecture, the current paper proposes the structure of a reactive architecture for gamified learning systems, which integrates intelligence, interaction, and multi-channel personalized dialogs in a resilient, flexible, and message-driven architecture.

### 5.4. Building Chatbots for Personalized and Contextual Feedback

**Figure 1:**
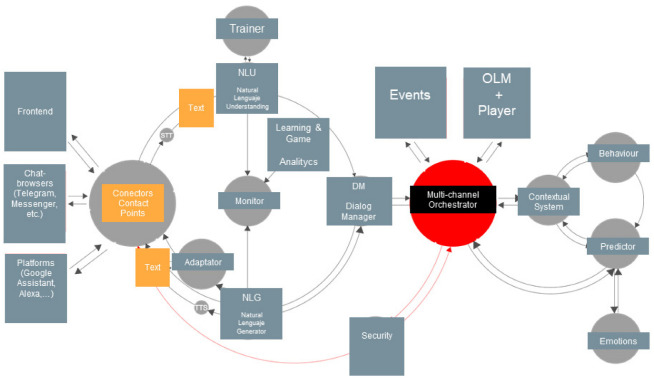
Chatbot Architecture Proposal showing the conversation cycle.

Therefore, building educational chatbots to enhance education is challenging to enhance current educational frameworks. Nevertheless, making an educational chatbot can be complex. Consequently, it is necessary to divide its design into several functional modules using different solutions and other methods (Figure 1).

- The contact points (connectors) are the entry points of information from the user by several channels.
- Then, depending on the format of entries (text or speech), the reader is transformed by S.T.T. or is just text.
- Once we have the text, the N.L.U subsystems look for intents and entities to understand the meaning of entries.
- The text entries feed the trainer to improve the understanding process.
- The dialog manager designs the possible conversation considering the previous understanding by the N.L.U.
- Then, the N.L.G. generates the answer, which can be in different formats (video, audio, images, buttons, etc.) or standard text. In this process, the TTS can be applied if it is required.
- The orchestrator influences this conversation cycle. Depending on the context and status of the conversation, the orchestrator can generate different outcomes in the dialog manager. The orchestrator depends on other events, access to APIs to which it can be connected, and the context systems.
- The system context is influenced by user behavior, the emotional status of the user, and different predictions.
- Security is another layer in the conversation cycle and a fundamental part of the orchestrator process.

(a) Intelligence Module

At the core of the chatbot, messages, answers, and questions must be built and designed following a rational and logical structure, and the chatbot’s objectives at each moment are achieved. Maintaining context over some time is a crucial requirement for dialog systems. There are different approaches to building conversational (dialog) chatbots. From the AI perspective, unsupervised learning is the most natural context concerning the type of information available. At the same time, one of the most challenging ways of deploying the architecture [60]. In this approach, the model can be trained from historical chat log data (transcripts) without human labeling directly. For example, we can mention the application of a sequence-to-sequence unsupervised recurrent neural network deep learning framework (seq2seq R.N.N.) [61]. On the other hand, other systems use methods based on recovery and Google’s machine learning platforms (Google Assistant). Applying this model, we find different types of chatbots: (a) vertical (closed-domain chatbots focused on particular vertical applications) or (b) horizontal chatbots (open-domain chatbots such as Siri, Google Assistant, or Alexa).

(b) Interaction Module

A different part of the chatbot design focuses on how the user interacts with the system. Many chats have a voice message feature; even speech recognition is considered an AI-hard problem that has recently reached acceptable accuracy levels. Although the essential bidirectional communication with a chatbot is through text, it is not the only possible type of interaction. It can also be through buttons, selectors, images, videos, etc. Frontends, platforms, or chat browsers have different formats to not only receive but also issue information: in a Google Home or Alexa-style system, it is possible to request something by voice and see the result on TV, for example. To achieve this, it must have drivers that allow, depending on the channel through which we interact with the user, to bring this enriched content. Therefore, all these processes must be orchestrated.

(c) Multi-channel Integration: The orchestrator

We have analyzed different natural language recognition systems (N.L.U.s), such as Dialogflow (formerly Google’s api.ai), LUIS (Microsoft), wit.ai (Facebook), I.B.M. Watson conversation, Lex (Amazon), etc. In this analysis, we have taken into account the accuracy of AI, training capacity, management of entities, contexts, connectors, etc. ChatBrowsers are applications in the client (usually multi-device) that allow access to chat systems. It can be Frontends, Apps, Telegram, Facebook messenger, Enterprise WhatsApp, Slack, Matrix, etc. It is important to use all these channels and provide an adequate service to have adapters that make it possible to send the information in the appropriate format to each one. So, the interaction of users on several different channels must be orchestrated. The orchestrator is the element that will support, for example, continuous interaction with a user through the various media, always maintaining a coherent and unified context in different areas. A script and a dialogue control system must be created to allow a chatbot to converse with the user. It will appropriately support interaction and response.

(d) Dialog Personalization: Reactive Architecture

Due to the non-linear and concurrent nature of the user’s interactions with the chatbot through different channels, using reactive architectures is essential, especially if you want to achieve a flow of conversation as naturally as possible. Reactive architectures are described as resilient, elastic, and message-driven. Actor-based systems are designed to be fault-tolerant and scale elastic through messages based on events and service load. Due to these event-oriented messaging systems, interoperability between different elements is ensured. For example, changing the web page can cause the chatbot to proactively ask for some information, if necessary. Due to the degree of concurrence required, a powerful chatbot must be equipped with a system that supports it. Using the model of actors and vibrant data structures is vital for developing such complex systems. Depending on the contexts, interactions, and experiences stored, it will be possible to analyze and apply all this information to make predictions using learning analytics and machine learning to improve the system and provide better service. Using cognitive explorers, it will be possible to structure the knowledge generated and give access (internal and external) to it through different channels, including chatbots. Using tools to analyze feelings, user behavior and other patterns detected, the user experience can be optimized.

## 6. Validation

To test the gamification design in an educational chatbot, we have created a prototype using the SnatchBot platform. The platform was selected among others because it is effortless to build a chatbot and has different templates you can reuse and adapt to your needs.

Additionally, this platform was selected because it is an omnichannel platform. SnatchBot’s tools cover the entire lifecycle of a bot, from development and testing to deployment, publishing, hosting, tracking, and monitoring and include N.L.P., machine learning, and speech recognition. The platform provides robust administrative features and adaptive, enterprise-grade security that meets all regulatory mandates.

**Figure 2:**
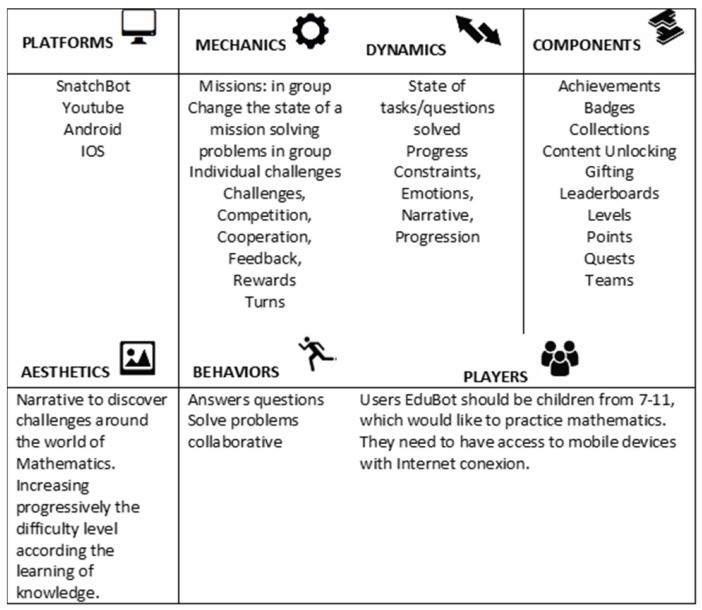
Gamification model canvas for our prototype. Adapted from Jimenez (2013).

**Figure 3:**
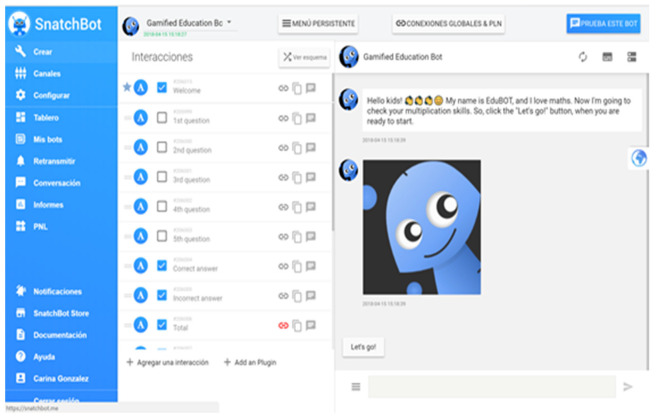
An example of an educational bot to practice mathematics.

In our case, our bot prototype aims to encourage kids to practice multiplication tables playfully. In the design of the gamification processes, we used an adapted version of the Gamification Model Canvas framework [62] (Figure 2 and Figure 3).

**Figure 4:**
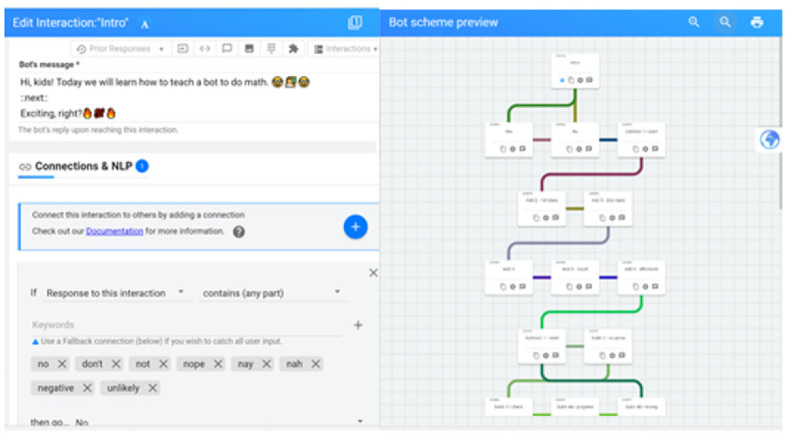
An example of a conversation schema flow and interaction definitions using connections and NLP.

The bot’s conversation consists of a set of messages, not only text messages but also images, audio, and video. Replies can be quick buttons of the persistent menu. The logic of the conversations is defined in the bot schema or dialog node tracking. Each interaction allows us to determine a personalized gamification’s mechanics, dynamics, and components. According to the user’s answers (student–player) and their preferences, the conversation will develop customized (Figure 4).

The prototype was validated by subject matter and technical experts because the chatbot was designed for a specific domain. This validation can be helpful to have subject matter experts review and test the chatbot to ensure that it provides accurate and relevant information. Additionally, the experts were technical experts that could review the chatbot’s code and infrastructure to ensure that it is well engineered and scalable. So, the process of data collection consisted of identifying and selecting the experts involved and conducting semistructured interviews. The instrument used in the validation was a semistructured interview. In this sense, we have interviewed five developer bot experts from ages 27 to 48 years old with more than one year of experience building chatbots. We present some of the great questions and answers of the experts:

What are the critical success factors and implementation barriers in educational chatbots?: Several critical issues were identified. First, individual differences influence the interaction with chatbots and are directly related to the learning process. Attitudes, skills, emotions, and personality can directly affect the chatbot’s behavior and the correspondent effect on student learning.

What elements of the architecture could be excluded or included?: Social, cultural, and individual backgrounds should be considered to generate a more natural dialog between the chatbot and users, not only the context and the learning goals.

What success factors should be considered when implementing gamification in chatbots?: Self-efficacy and self-regulated skills have a positive impact on the autonomy of the students with their learning. Gamified chatbots can support the training of these skills in a personalized and engaging way. Learning outcomes depend strongly on the student’s characteristics, and their motivation is the key. Gamifying the learning process in a customized way can contribute to success. 

In your opinion, what would be the main barriers to gamification in chatbots?: Many chatbots are tree-based dialogs previously designed by the developer. In this kind of chatbot, to avoid the frustration caused by wrong answers, a large dataset is needed. Additionally, several elements of gamification are challenging to implement in dialog-based chatbots, such as those related to social issues.

## 7. Conclusions

In this paper, we have presented a complete proposal of a tool for personalized gamification for learning using a chatbot architecture.

The first contribution of the paper is the Proposal of a joint open student–player model. The lack of such a collaborative model has been traditionally a handicap of this type of system. Additionally, a general system architecture, including a dynamic machine learning system validation, has been described to increase the system’s adaptability to multiple digital applications while keeping the model simple. The mentioned open model is one of the modules of our architecture. This modular architecture includes a game learning analytics and personalization module and another to collect contextual information from the user for personalization. Finally, the gamification engine is designed to be responsible for real-time personalization features.

A reactive chatbot architecture has been implemented based on the proposed general architecture definition. Building blocks for the proposed implementation are described. This includes the multi-channel integration: the orchestrator. The orchestrator appropriately supports interaction and response based on interactions with digital sources.

The validation of the architecture has implied the implementation of a prototype using the SnatchBot platform. The bot has the goal of encouraging kids to practice multiplication tables. Expert opinions have been gathered to complete the validation.

The key results of the validation have been to identify critical success factors and implementation barriers for both objectives, the personalized implementation of educational chatbots, and their gamification. Among the discovered factors is the recommendation to include social, cultural, and individual backgrounds for the personalization and the support of gamified chatbots on self-efficacy and self-regulated skills, which positively impact the students’ autonomy. Some potential barriers have been described, such as the need for an extensive collection of data and the influence of attitudes, skills, emotions, and personality in the chatbot behavior affecting student learning.

In future work, the research should be conducted further in different directions. Firstly, the prototype should be extended to allow for longitudinal studies of the interaction with the students. Not only into one specific field but also making cross-disciplinary evaluation possible. Secondly, a modular prototype extension should be allowed to create dialogs and gamified experiences. Thirdly, an overall assessment of the prototype should be designed, including cross-cultural and diversity evaluation of the system should be analyzed. Finally, more sophisticated NLP AI new-generation technologies should be explored for integration to enrich the user experience with the system. 

## Data Availability

Not applicable.
